# Supplementary material for: Near Infrared Spectroscopy for Prediction of Yeast and Mould Counts in Black Soldier Fly Larvae, Feed and Frass: A Proof of Concept
Source: Sensors (Basel). 2023 Aug 4;23(15):6946. doi: 10.3390/s23156946 (PMC10422329; doi:10.3390/s23156946)
Supplement: Supplementary file 1 [file sensors-23-06946-s001.zip › sensors-2499737-supplementary.pdf]

Supplementary figures

Figure S1. Near infrared raw spectra of all samples (feed, frass and larvae) analysed.

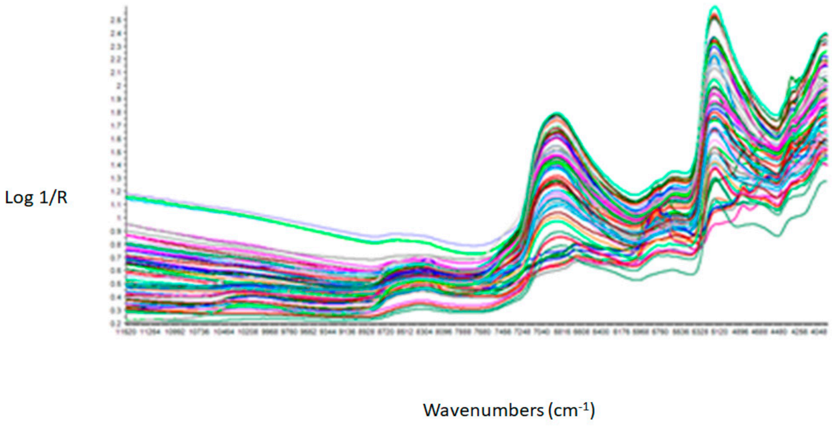

Figure S2. Near infrared baseline corrected spectra of all samples (feed, frass and larvae) analysed.

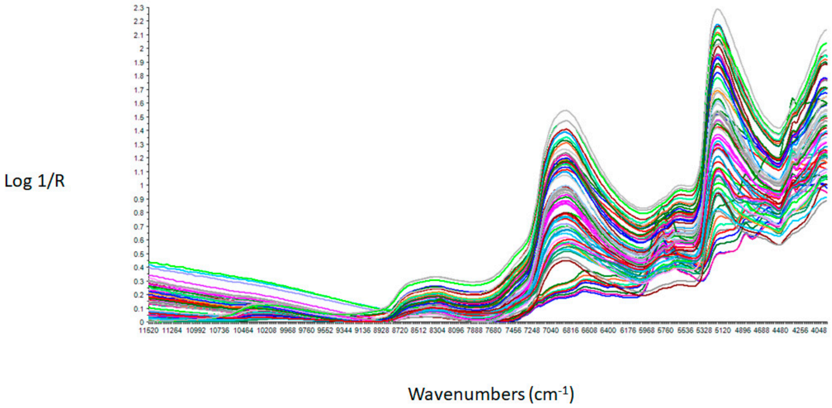

Figure S3. First derivative of the NIR spectra of all samples (feed, frass and larvae) analysed.

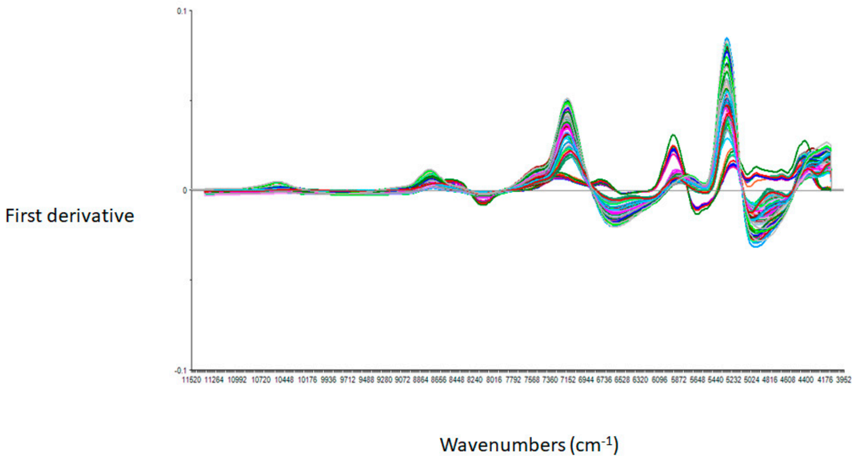

Figure S4. Second derivative of the NIR spectra of all samples (feed, frass and larvae) analysed.

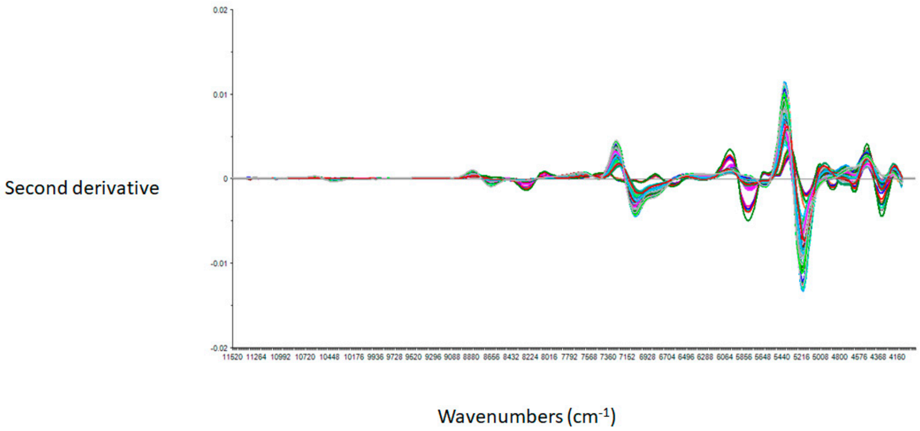

Second derivative
0.02
0.01
0
-0.01
-0.02
11520 11264 10992 10720 10448 10176 9936 9728 9520 9296 9088 8880 8656 8432 8224 8016 7792 7568 7360 7152 6928 6704 6496 6288 6064 5856 5648 5440 5216 5008 4800 4576 4368 4160
Wavenumbers (cm-1)
